# The feasibility of proteomics sequencing based immune-related prognostic signature for predicting clinical outcomes of bladder cancer patients

**DOI:** 10.1186/s12885-022-09783-y

**Published:** 2022-06-20

**Authors:** Liren Jiang, Siteng Chen, Qi Pan, Jun Zheng, Jin He, Juanjuan Sun, Yaqin Han, Jiji Yang, Ning Zhang, Guohui Fu, Feng Gao

**Affiliations:** 1 Pathology Center, Shanghai General Hospital, Shanghai Jiao Tong University School of Medicine, No. 100, Hai Ning Road, Shanghai, 200080 China; 2 Department of Urology, Renji Hospital, Shanghai Jiao Tong University School of Medicine, Shanghai, China; 3 Department of Urology, Shanghai General Hospital, Shanghai Jiao Tong University School of Medicine, Shanghai, China; 4 Department of Urology, Ruijin Hospital, Shanghai Jiao Tong University School of Medicine, No. 197 Ruijin 2nd Road, Shanghai, 200020 China; 5 Key Laboratory of Cell Differentiation and Apoptosis of Chinese Ministry of Education, Institutes of Medical Sciences, Shanghai Jiao Tong University School of Medicine, Shanghai, China; 6 Shanghai Key Laboratory of Gastric Neoplasms, Shanghai Institute of Digestive Surgery, Ruijin Hospital, Shanghai Jiao Tong University School of Medicine, No. 280, South Chong-Qing Road, Shanghai, 200025 China

**Keywords:** Immune-related, Prognostic signature, Bladder cancer, PD-1, PD-L1

## Abstract

**Background:**

Bladder cancer (BCa) shows its potential immunogenity in current immune-checkpoint inhibitor related immunotherapies. However, its therapeutic effects are improvable and could be affected by tumor immune microenvironment. Hence it is interesting to find some more prognostic indicators for BCa patients concerning immunotherapies.

**Methods:**

In the present study, we retrospect 129 muscle-invasive BCa (MIBC) patients with radical cystectomy in Shanghai General Hospital during 2007 to 2018. Based on the results of proteomics sequencing from 9 pairs of MIBC tissue from Shanghai General Hospital, we focused on 13 immune-related differential expression proteins and their related genes. An immune-related prognostic signature (IRPS) was constructed according to Cancer Genome Atlas (TCGA) dataset. The IRPS was verified in ArrayExpress (E-MTAB-4321) cohort and Shanghai General Hospital (General) cohort, separately. A total of 1010 BCa patients were involved in the study, including 405 BCa patients in TCGA cohort, 476 BCa patients in E-MTAB-4321 cohort and 129 MIBC patients in General cohort.

**Result:**

It can be indicated that high IRPS score was related to poor 5-year overall survival and disease-free survival. The IRPS score was also evaluated its immune infiltration. We found that the IRPS score was adversely associated with GZMB, IFN-γ, PD-1, PD-L1. Additionally, higher IRPS score was significantly associated with more M2 macrophage and resting mast cell infiltration.

**Conclusion:**

The study revealed a novel BCa prognostic signature based on IRPS score, which may be useful for BCa immunotherapies.

**Supplementary Information:**

The online version contains supplementary material available at 10.1186/s12885-022-09783-y.

## Introduction

Bladder cancer (BCa) is the fourth most common cancer in men, with estimated 81,400 new incidence and 17,980 deaths in the United States in 2020 [1]. Currently, BCa can be divisible into non-muscle-invasive BCa (NMIBC) and muscle-invasive BCa (MIBC), with diverse clinical outcomes [2–4]. The 5-year overall survival of NMIBC patients is over 99% whereas that of MIBC patients drops down dramatically [3, 4]. It is about 50% of MIBC patients that still appear local recurrence or distant metastasis after radical cystectomy and chemotherapy [4, 5]. Classical and traditional prognostic indicators for BCa patients are mainly TNM staging and pathological differentiation [6]. Nevertheless, these parameters may be insufficient for current clinicians’ demands [7, 8]. Hence, it becomes necessary to find some more practical prognostic signatures besides traditional TNM staging and histopathological features [6, 9].

Through the process of malignancy proliferation, cancer cells gradually accelerate the escape from the monitor of normal immune system, resulting in progression and metastasis [10, 11]. Inhibitory immune cells are recruited by cancer cells in the tumor microenvironment to suppress the anti-tumor immune response, which are caused by immune checkpoints and some other cytokines [10, 12, 13]. Currently, some immune-checkpoint inhibitors targeting programmed death-1 (PD-1) and programmed death-ligand 1 (PD-L1) are approved by Food and Drug Administration (FDA) to treat locally advanced or metastatic platinum-ineligible MIBC patients [14]. However, these immune-checkpoint inhibitor targeted drugs can only benefit limited MIBC patients [15–17]. Moreover, around 10% MIBC patients treated with immune-checkpoint inhibitors may have hyper-progressive disease [18, 19]. The PD-1/PD-L1 related immunotherapies for MIBC patients are still not satisfactory [20].

In this study, based on the results of proteomics sequencing from 9 pairs of MIBC patient samples, we focused on the immune-related differential proteins with prognostic values. The immune-related prognostic signature (IRPS) was constructed based on Cancer Genome Atlas (TCGA) cohort and verified by ArrayExpress (E-MTAB-4321) cohort and Shanghai General Hospital (General) cohort. A total of 1010 BCa patients were involved in the study. High IRPS score was also correlated with the remodeling of the immune microenvironment.

## Materials and methods

### Patient samples and ethics statement

In General cohort, totally 129 MIBC patient samples were included in the study. All the involved patients undergone radical cystectomy in Shanghai General Hospital during 2007 to 2018. Patients with concurrent other cancers, autoimmune diseases or HIV infections were excluded in the study. The study was conducted according to the Helsinki Declaration and was approved by the Human Ethics Committee of Shanghai General Hospital. The clinicopathological features of the 129 MIBC patients in General cohort were shown in Table 1.

**Table 1:** The clinicopathological features in all cohorts

Clinical characteristics	TCGA cohort (*n* = 405)	E-MTAB-4321 cohort (*n* = 476)	General cohort (*n* = 129)
Age	69 (34 ~ 90)	69 (24 ~ 96)	67 (47 ~ 89)
Gender			
Female	106	109	20
Male	299	367	109
Stage			
I	2	460	0
II	129	16	76
III	139	0	53
IV	133	0	0
Unknown	2	0	0
T			
Ta	0	345	0
Cis	0	3	0
T1	3	112	0
T2	118	16	77
T3	192	0	27
T4	58	0	25
Unknown	34	0	0
N			
N0	235	/	122
N1	46	/	5
N2	75	/	1
N3	7	/	1
Unknown	42	/	0
M			
M0	195	/	129
M1	11	/	0
Unknown	199	/	0

In addition, a total of 405 BCa patient samples from TCGA dataset and 476 BCa patient samples from E-MTAB-4321 dataset were used in the study [21]. All the enrolled sample data were with complete gene expression and clinical data.

### Proteomics sequencing

A total of 9 pairs of MIBC samples from Shanghai General Hospital were conducted liquid chromatography-mass spectrometry (LC–MS). Each pair included BCa and normal bladder tissue from each individual patient. All the 9 pairs of samples were classified into two groups based on survival. The 3 MIBC patients were survived for 5 years and were considered as good prognosis. The 6 MIBC patients, regarded as poor prognosis, were passed away. All the 9 male MIBC patients were at BCa stage of T2N0M0 and among 62 to 70 years old. The abovementioned MIBC patients were from the institutional cohort (General cohort, *n* = 129, after excluding the 9 MIBC samples, which were used for LC/MS analysis). Each formalin-fixed and paraffin-embedded sample was used the amount of 10 consecutive section slides with 10 μm thickness. Afterwards, paraffin was removed by xylene, heptane and methanol washes. Samples were treated by 7 M urea, 2 M thiourea, 1 M ammoniumbicarbonate as well as protease and phosphatase inhibitor cocktails. Then samples were heated to 95 °C for 30 min and incubation at 60 °C for 2 h, followed by sonication and centrifugation. Protein concentration was determined by Bradford assay (Bio-Rad). Peptides were desalted and dried on Strata X C18 SPE columns (Phenomenex). Each 100 μg sample was dissolved in 30 μl dissolution buffer and labeled at room temperature with one iTRAQ reagent solution for 2 h, followed by 30 min with 100 μL of Milli-Q water. All the samples were then mixed into one tube and dried. We used the Suveyor high performance liquid chromatography system (Thermo Fisher Scientific) and LTQ-Orbitrap instrument (Thermo Fisher Scientific) to perform the reverse-phase high performance liquid chromatography separation. The flow rate of the reverse-phase high performance liquid chromatography separation was 250 nl/min. We used data-dependent mode to store data. All raw MS files were processed by MaxQuant software and based on the UniProt human database. We set 1% false discovery rate for protein and peptide identification. The minimum peptide length was set as seven amino acids. The fragment ion tolerance was set as 20 ppm. We set carbamidomehtylation of cysteine residues as fixed modification. Variable modifications included oxidation of methionine and N-carbamylation. All the detected proteins were classified by their molecular functions through Gene ontology (GO) enrichment analysis and Kyoto Encyclopedia of Genes and Genomes (KEGG) pathway analysis [22].

### Construction of the prognosis model for BCa

A total of 13 differentially expressed immune-related genes from proteomics sequencing results were selected as candidate genes. These 13 selected genes were conducted least absolute shrinkage and selection operator (LASSO) cox regression analysis via *glmnet* package. Afterwards, 7 genes with prognostic values were found and their coefficients were calculated based on TCGA cohort. The number of lambda in LASSO was set up to 1000. The IRPS score was formulated in the following:

$$\mathrm{IRPS score }={\sum }_{i=1}^{n}(\mathrm{Coefi}*\mathrm{Ri})$$

Coefi represented the coefficient of each the 7 focused genes in this study and Ri stood for their corresponding mRNA expressions. The detailed calculation of IRPS score was: IRPS score = STAT3 * 0.30218322 + TGFB1 * 0.16910315 + CTSG * 0.05205169—NFKB1 * 0.34611795—SNRPD2 * 0.15818733—PD-1 * 0.09569928—TAP1 * 0.01611763. The IRPS was further validated in the E-MTAB-4321 cohort and the General cohort, covering 605 BCa patients. Both the cut-off values of IRPS score in the two cohorts were set as the median score of each cohort.

#### Immunohistochemistry (IHC)

MIBC patient samples from Shanghai General Hospital were stained with anti-Cathepsin G (Affinity, 1:100), anti-NF-κB1 (Affinity, 1:100), anti-STAT3 (Affinity, 1:100), anti-TGF-β1 (Affinity, 1:100), anti-SNRPD2 (Affinity, 1:100), anti-TAP1 (Affinity, 1:100) and anti-PD-1 (Affinity, 1:100). All the primary antibodies were incubated overnight at 4℃ and secondary antibodies were incubated for 90 min at room temperature. Slides were visualized with 3, 3′-diaminobenzidine and hematoxylin.

#### Immune infiltration analysis

We applied the CIBERSORT to estimate the abundances of 22 types of immune cells [23]. Here in this study, the mean expression values of PD1, PD-L1, CTLA-4, LAG-3 and TIM-3 were regarded as immune checkpoint (ICK) score; whereas the mean expression values of granzyme B (GZMB), interferon-γ (IFN-γ) and perforin (PRF1) were set as Effector score [24]. We obtained the lymphocyte infiltration signature score from CRI iAtlas [25].

### Statistical analysis

In this study, the differentially expressed proteins between two different prognosis groups were analyzed by linear models for microarray data (limma). We used either a two-tailed Mann Whitney U test or a one-way analysis of variance to analyze continuous variants. The prognostic values of immune-related genes were analyzed by Cox regression analysis. Kaplan–Meier curves of overall survival (OS) and disease-free survival (DFS) were analyzed by log-rank test. Correction analysis was carried out Spearman method. The statistical analysis and data visualization were achieved by R (3.6.2) and SPSS 24.0. A *P* value of < 0.05 was considered to be statistically significant. The flow chart diagram were shown in Supplementary Fig. 1.

## Results

### Investigation of differentially expressed immune-related proteins in MIBC patients

**Fig. 1:**
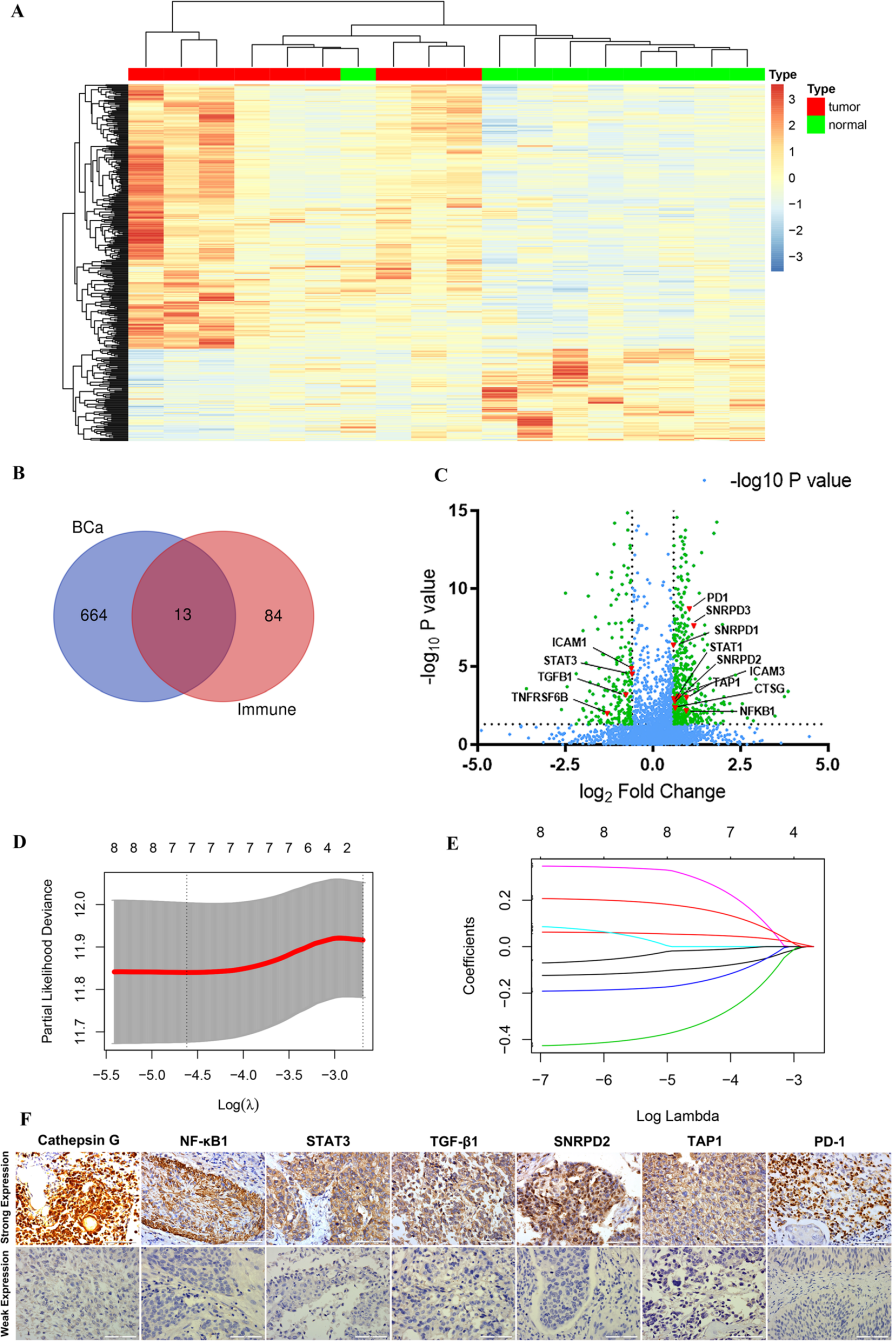
Identification of immune-related genes with prognostic values. **A** The heatmap of LC–MS from 9 pairs of MIBC patients with all the differential expression proteins between two different prognosis groups. **B** A total of 13 out of 677 differentially expressed proteins were classified as immune-related proteins. **C** The Volcano plot result of all the 677 differential expression protein. **D** The LASSO analysis of the 13 immune-related genes. **E** The profile of coefficients of the 7 immune-related genes. **F** The illustration of expression levels of 7 immune-related proteins in General cohort. LC–MS: liquid chromatography-mass spectrometry. LASSO: least absolute shrinkage and selection operator

In order to identify differential expression of immune-related proteins among cancer cells and normal bladder cells, we performed a LC–MS analysis based on 9 pairs of samples from MIBC patients resected in Shanghai General Hospital during 2007 to 2018 (Fig. 1A). Between the two different prognosis groups, there were totally 677 differentially expressed proteins, with a *P* value < 0.05 and |fold change|> 1.5 (Log2(fold change) > 0.584). Considering the GO and KEGG analysis, we found 97 immune-related proteins (Fig. 1B, C). Interestingly, 13 out of 97 immune-related proteins were also detected as differential expression proteins in MIBC patients, including ICAM1, STAT3, TGF-β1, TNFRSF6B, SNRPD3, STAT1, SNRPD2, ICAM3, TAP1, Cathepsin G, PD-1 and NF-κB1 (Fig. 1B, C). Therefore, we decided to further investigate the prognostic values of the 13 proteins related genes.

### Construction of IRPS based on the BCa patients in TCGA cohort

**Fig. 2:**
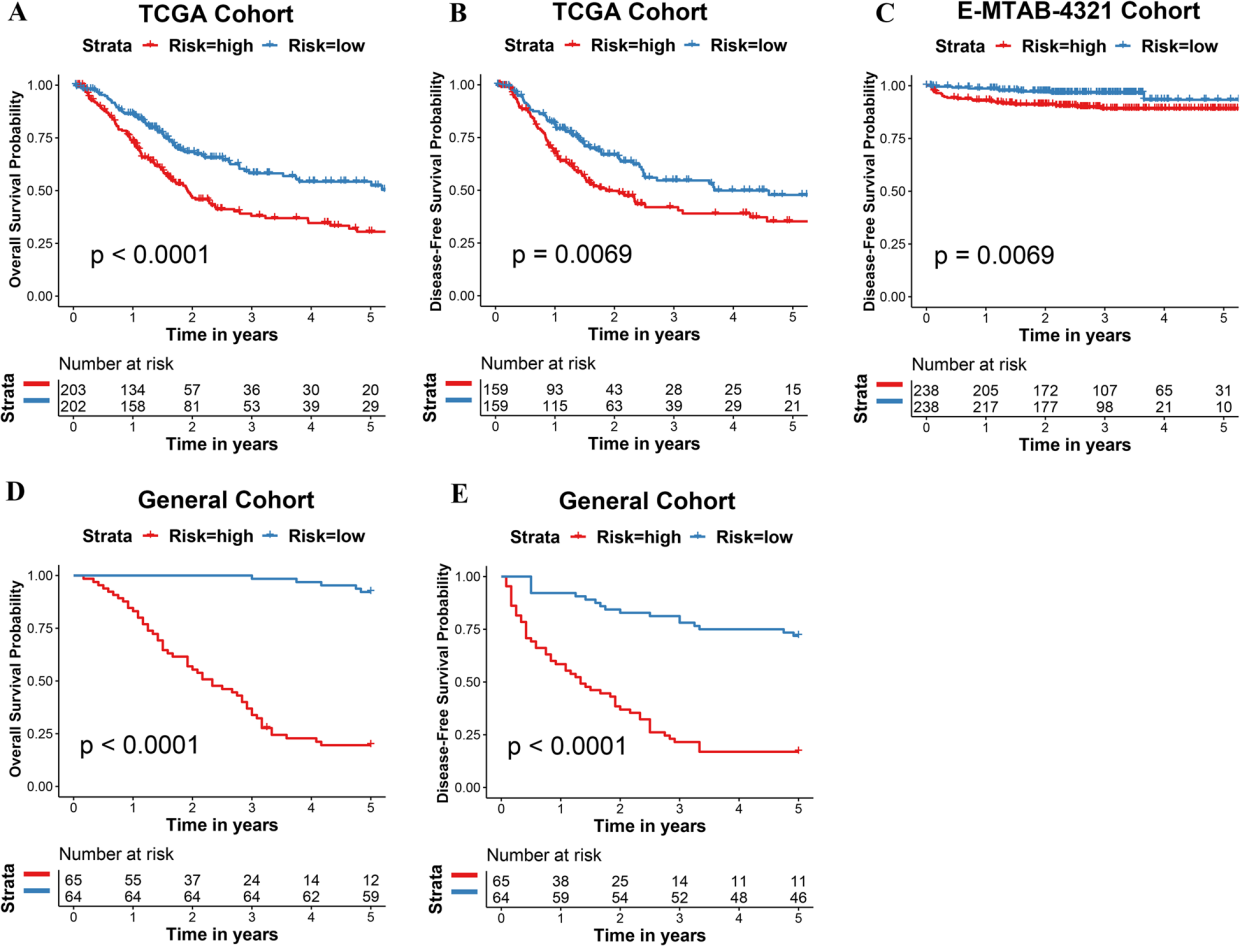
Kaplan–Meier survival analysis among 3 cohorts. **A** The OS of MIBC patients in TCGA cohort. **B** The DFS of MIBC patients in TCGA cohort. **C** The DFS of BCa patients in E-MTAB-4321 cohort. **D** The OS of MIBC patients in General cohort. **E** The DFS of MIBC patients in General cohort. OS: overall survival. DFS: disease-free survival

**Fig. 3:**
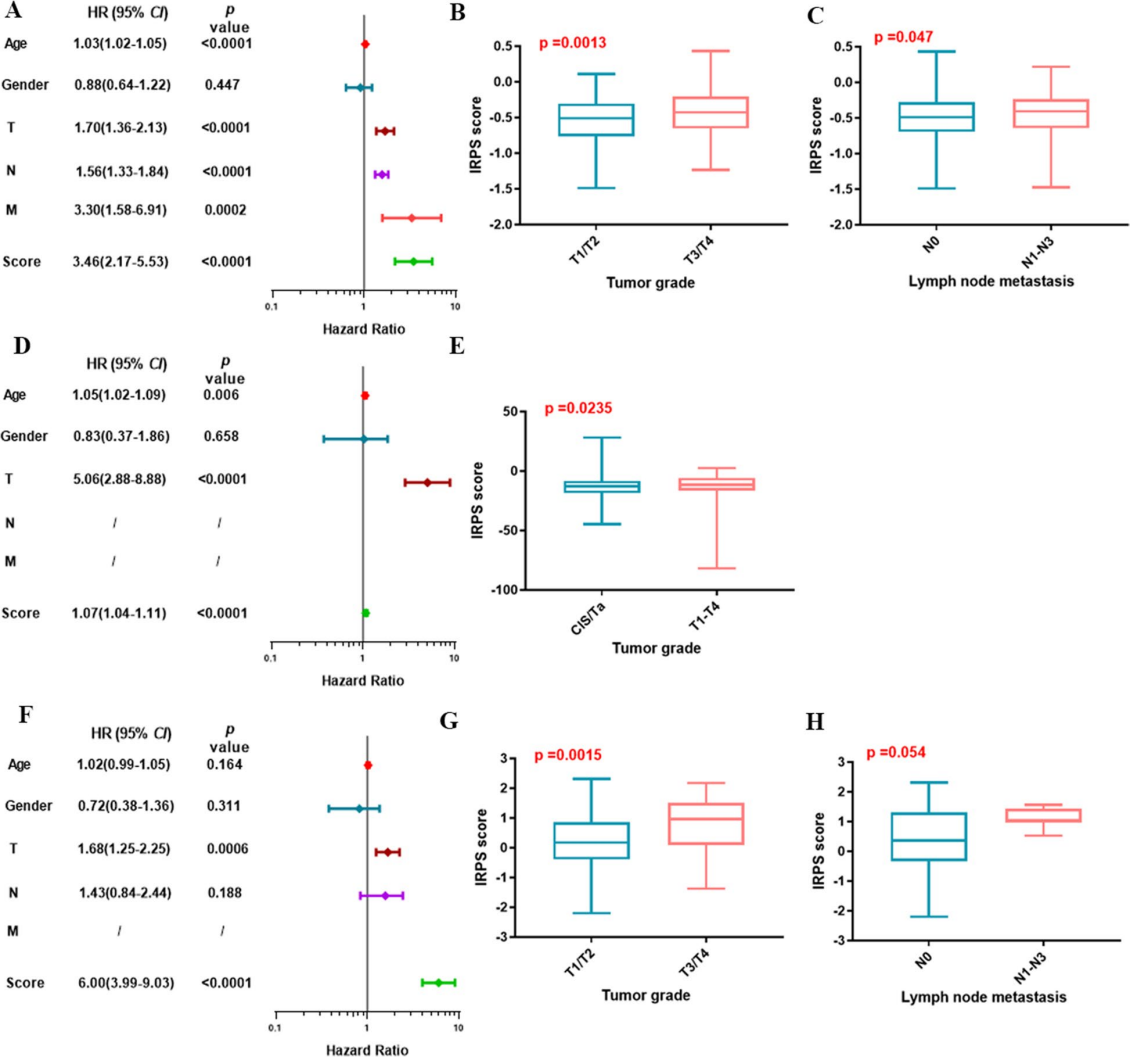
Correlation of IRPS score and clinicopathological features among 3 cohort. **A** Cox regression analysis of IRPS score and clinicopathologic features for patients’ OS in TCGA cohort. **B** The correlation of IRPS score and tumor grade in TCGA cohort. **C** The correlation of IRPS score and lymph node metastasis in TCGA cohort. **D** Cox regression analysis of IRPS score and clinicopathologic features for patients’ DFS in E-MTAB-4321 cohort. **E** The correlation of IRPS score and tumor grade in E-MTAB-4321 cohort. **F** Cox regression analysis of IRPS score and clinicopathologic features for patients’ OS in General cohort. **G** The correlation of IRPS score and tumor grade in General cohort. **H** The correlation of IRPS score and lymph node metastasis in General cohort. OS: overall survival. DFS: disease-free survival

All the 13 immune-related genes were undergone LASSO-Cox regression analysis in the TCGA cohort (Fig. 1D, E). Interestingly, a total of 7 immune-related genes were shown their statistical significance (*P* < 0.05) in predicting the clinical outcomes of BCa patients, including Cathepsin G, NF-κB1, STAT3, TGF-β1, SNRPD2, TAP1 and PD-1. Subsequently, the IRPS was constructed based on the 7 genes in TCGA dataset. Meanwhile, the IRPS score was established according to the formulation shown in 2 section. In TCGA cohort, 405 BCa patients were classified into IRPS high and low groups based on the cut-off IRPS value. BCa patients with IRPS high score had significantly poorer 5-year OS (*P* < 0.0001) with hazard ratio of 3.46 (Figs. 2A, 3A). Whereas, BCa patients with IRPS high score were associated with a worse 5-year DFS (*P* = 0.0069) (Fig. 2B). Additionally, patients with T3/T4 stages and lymph node metastasis were associated with higher IRPS score (*P* = 0.0013 and *P* = 0.047, respectively) (Fig. 3B, C).

### Verification of IRPS among BCa patients in E-MTAB-4321 cohort

We further explored whether the IRPS can be applied as a prognostic model in different dataset. As a result, the IRPS was verified in E-MTAB-4321 cohort. A total of 476 BCa patients were enrolled in the E-MTAB-4321 dataset, including 460 NMIBC and 16 MIBC patients. As expected, high IRPS score BCa patients also showed a statistically significant poorer 5-year DFS outcomes in E-MTAB-4321 cohort (*P* = 0.0069) with hazard ratio of 1.07 (Figs. 2C, 3D). Moreover, it was also indicated that Cis/Ta-stage BCa patients were correlated with lower IRPS score (*P* = 0.0235) (Fig. 3E).

### Verification of IRPS of MIBC in general cohort

We further verified the effectiveness of IRPS in predicting clinical outcomes of MIBC patients in General cohort. The hazard ratio value of each protein was illustrated in Table 2. The illustration of expression levels of the 7 immune-related proteins were shown in Fig. 1F. MIBC patients in General cohort were subdivided according to the mean value of IRPS score. Consistently, the 5-year OS and DFS of MIBC patients with high IRPS score dropped significantly (*P* < 0.0001 and *P* < 0.0001, respectively) (Fig. 2D, E). The hazard ratio of high IRPS score and 5-year OS was 6.00 (Fig. 3F). Also, T3/T4 stages and lymph node metastasis were correlated with higher IRPS score (*P* = 0.0013 and *P* = 0.047, respectively) (Fig. 3G, H). These results indicated IRPS an independent prognostic indicator for MIBC.

**Table 2:** The results of hazard ratio values for each protein in General cohort

Protein	Hazard Ratio	95% Confidence Interval	*p* value
NFKB1	0.69	0.58–0.82	< 0.001
STAT3	1.00	0.82–1.22	0.994
CTSG	1.47	1.26–1.71	< 0.001
TGFB1	1.49	1.23–1.81	< 0.001
SNRPD2	0.60	0.52–0.70	< 0.001
TAP1	0.64	0.55–0.74	< 0.001
PDCD1	0.61	0.51–0.72	< 0.001

### Correlation of IRPS score and the remodeling of the immune microenvironment

**Fig. 4:**
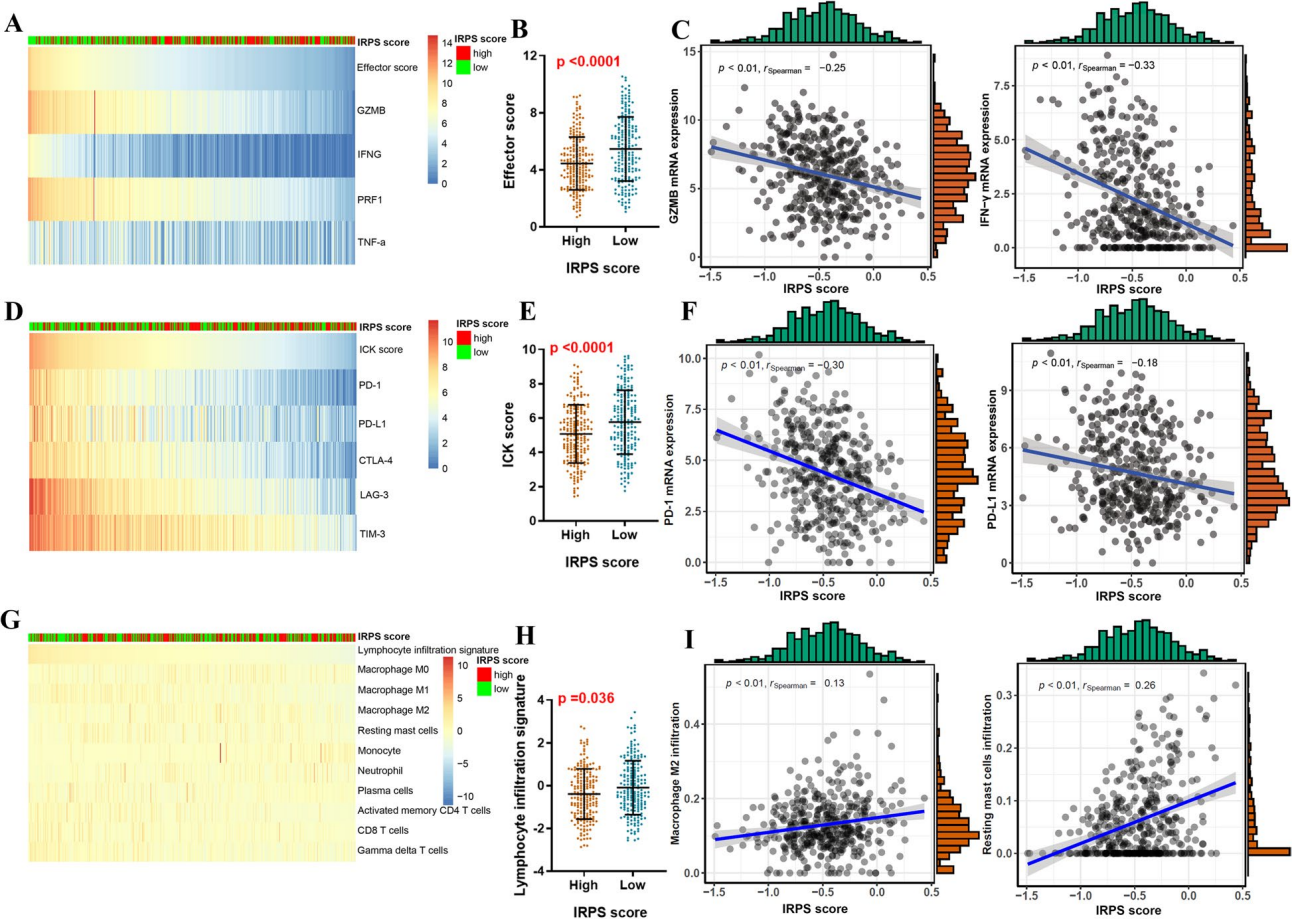
Correlation of IRPS score and the remodeling of the immune microenvironment. **A** Heatmap of IRPS score and the expression of effector molecules. **B** Evaluation of the correlation among IRPS score and Effector score. **C** Correlation of IRPS score and GZMB or IFN-γ expression. **D** Heatmap of IRPS score and the expression of ICK molecules. **E** Evaluation of the correlation among IRPS score and ICK score. **F** Correlation of IRPS score and PD-1 or PD-L1 expression. **G** Heatmap of IRPS score and the infiltration of 22 types of immune cells. **H** Evaluation of the correlation among IRPS score and lymphocyte infiltration signature score. **I** Correlation of IRPS score and M2 macrophage or resting mast cell infiltration

For the purpose of revealing the relationship between the IRPS score and the tumor immune microenvironment, we utilized immune infiltration analysis. The IRPS score was evaluated with the Effector score, ICK score and lymphocyte infiltration signature score (Fig. 4A, D, G). We found that Effector, ICK and lymphocyte infiltration signature score all significantly diminished in IRPS high score groups (*P* < 0.0001, *P* < 0.0001 and *P* = 0.036, respectively) (Fig. 4B, E, H). The IRPS score was adversely associated with GZMB, IFN-γ, PD-1, PD-L1 (Spearman’s R = -0.25, Spearman’s R = -0.33, Spearman’s R = -0.30 and Spearman’s R = -0.18; *P* < 0.01, *P* < 0.01, *P* < 0.01 and *P* < 0.01, respectively) (Fig. 4C, F). Additionally, higher IRPS score was significantly associated with more M2 macrophage and resting mast cell infiltration (Spearman’s R = 0.13 and Spearman’s R = 0.26; *P* < 0.01 and *P* < 0.01, respectively) (Fig. 4I). The complete analysis of IRPS score and immune cell infiltrations were shown in Supplementary Fig. 2.

## Discussion

It can be inferred that the potential immunogenity of BCa from the current utilization of PD-1/PD-L1 related immunotherapies [8, 20]. However, considering the immunosuppressive microenvironment in BCa tissue, it would be interesting to investigate the expression of immune-related genes in BCa [26]. Furthermore, the immune-checkpoint inhibitor targeted immunotherapies for MIBC patients need improvements [17]. Therefore, it would be intriguing to find some prognostic biomarkers concerning immune-checkpoint inhibitors for MIBC patients. In the present study, through proteomics sequencing, we established an IRPS for predicting the survival of BCa patients, especially MIBC patients, and analyzed the immune infiltration of IRPS score.

Among the 7 enrolled immune-related genes, Cathepsin G is a neutrophil serine protease and its interaction with receptor for advanced glycation end products can trigger neutrophil cytotoxicity to kill tumor cells [27, 28]. Studies demonstrate the prognostic value of Cathepsin G in oral squamous cell carcinoma patients [29]. It remains unclear that the detailed function of Cathepsin G in tumor immune response and its prognostic value for BCa patients. NF-κB1 belongs to NF-κB family, involved in regulating cellular proliferation, apoptosis, inflammation and tumor immune response [30]. The dysregulation of NF-κB1 results in hepatocellular carcinoma, acute lymphoblastic leukemia as well as breast cancer [30–32]. STAT3 is vital for vertebrate development while its mutations are associated with immunodeficiency, autoimmunity and cancer [33]. As previous studies reported, TGF-β1, a family member of TGF-β, plays a key role in the development and maturation of immune cells [34]. Drugs targeting TGF-β1 pathways for tumor immunotherapies are extremely difficult at present [34]. There are limited studies on SNRPD2, a small nuclear ribonucleoprotein D2 polypeptide [35]. Researchers have evaluated a prognostic signature with 13 key genes including SNRPD2 for hepatocellular carcinoma patients [35]. Detailed molecular mechanisms between SNRPD2 and cancer remain unknown. It is found that TAP1, antigen processing 1, is correlated with good prognosis in colorectal cancer and melanoma patients [36, 37].

Currently, the utility of immune-checkpoint inhibitors in malignancies has increasingly become a hot tendency in cancer treatment [38]. PD-1/PD-L1 is one of the most studied and clinically successful immune-checkpoint inhibitor drug targets [16]. Although it is reported that high levels of PD-L1 and PD-1 expressions in cancers suggest worse prognosis and advanced disease stages, only limited patients could benefit from PD-1/PD-L1 related immune-checkpoint inhibitor treatments [38]. Moreover, the criteria for PD-1/PD-L1 immunochemistry evaluation in BCa are still away from forming the consensus [16, 39]. Different pathologists may even gain a contradictory PD-1/PD-L1 evaluation result from the same pathological image. Therefore, it is necessary to find a convenient and comparatively reliable method for predicting immunotherapy responsiveness. The IRPS score in this study was statistically significant negatively associated with PD-1/PD-L1, which could be a potential clinical utilization of IRPS.

Intriguingly, IRPS score was correlated with distributions of some immune cells, especially positive correlation of macrophage M2 and adverse correlation of CD8 T cells. Currently, PD-1 positive CD8 T cells are the main targets for immune-checkpoint inhibitor therapies [40]. It is demonstrated that BCa patients with heavy CD8 T cell infiltration benefit more immune-checkpoint inhibitor therapies due to the higher accessibility of neoantigens, tumor-specific antigens encoded by mutated genes [38, 40]. Moreover, CD8 T cells can be enhanced by activated memory CD4 T cells [41, 42]. Macrophage M2 can increase immunosuppressive infiltration of Treg cells and decrease the immune-checkpoint inhibitor therapy responsiveness [43]. The correlation of IRPS score and distribution of immune cells implied potential mechanisms behind IRPS and tumor immune microenvironment of BCa.

Moreover, considering the performance of IRPS among TCGA, E-MTAB-4321 and General cohorts, we found that cohorts with higher MIBC patient ratio showed more obvious difference among IRPS high and low score groups. General cohort was consisted of only MIBC patients whereas E-MTAB-4321 cohort mostly was NMIBC patients. It can be suggested that IRPS performed better for MIBC patients. In addition, MIBC patients have much higher mortality and draw more medical attention from clinicians. Via IRPS evaluation, clinicians can briefly judge the prognosis of MIBC patients. At present, immune-checkpoint inhibitor targeted therapies for BCa patients are utilized to treat locally advanced or metastatic platinum-ineligible MIBC patients [14]. IRPS score could be a potential indicator for immunotherapies on MIBC patients.

There were also some limitations in this study. Firstly, the cut-off value was selected as the median value of each cohort for defining high and low IRPS score in this retrospective study. The best cut-off value for IRPS score shall be explored through further prospective studies. Secondly, the outcome and molecular biology of muscle-invasive and non-muscle invasive bladder cancer are considerably different, thus the non-MIBC cases in the E-MTAB-4321 cohort might cause potential bias. But we had also performed external validation in the General cohort with MIBC cases.

In conclusion, the present study constructed an IRPS based on LC–MS results and TCGA dataset. The IRPS was verified by E-MTAB-4321 cohort and General cohort. High IRPS score was correlated with worse clinical outcomes in BCa patients. The IRPS score showed its relevance with immune infiltration.

## Supplementary Information

**Additional file 1.** The workflow of the study.

**Additional file 2.** Correlation analysis of IRPS score and immune infiltration cells.

## Data Availability

All data generated or analyzed during this study are included in this published article.
