# Supplementary figures and images for: The feasibility of proteomics sequencing based immune-related prognostic signature for predicting clinical outcomes of bladder cancer patients
Source: BMC Cancer. 2022 Jun 20;22:676. doi: 10.1186/s12885-022-09783-y (PMC9210750; doi:10.1186/s12885-022-09783-y)

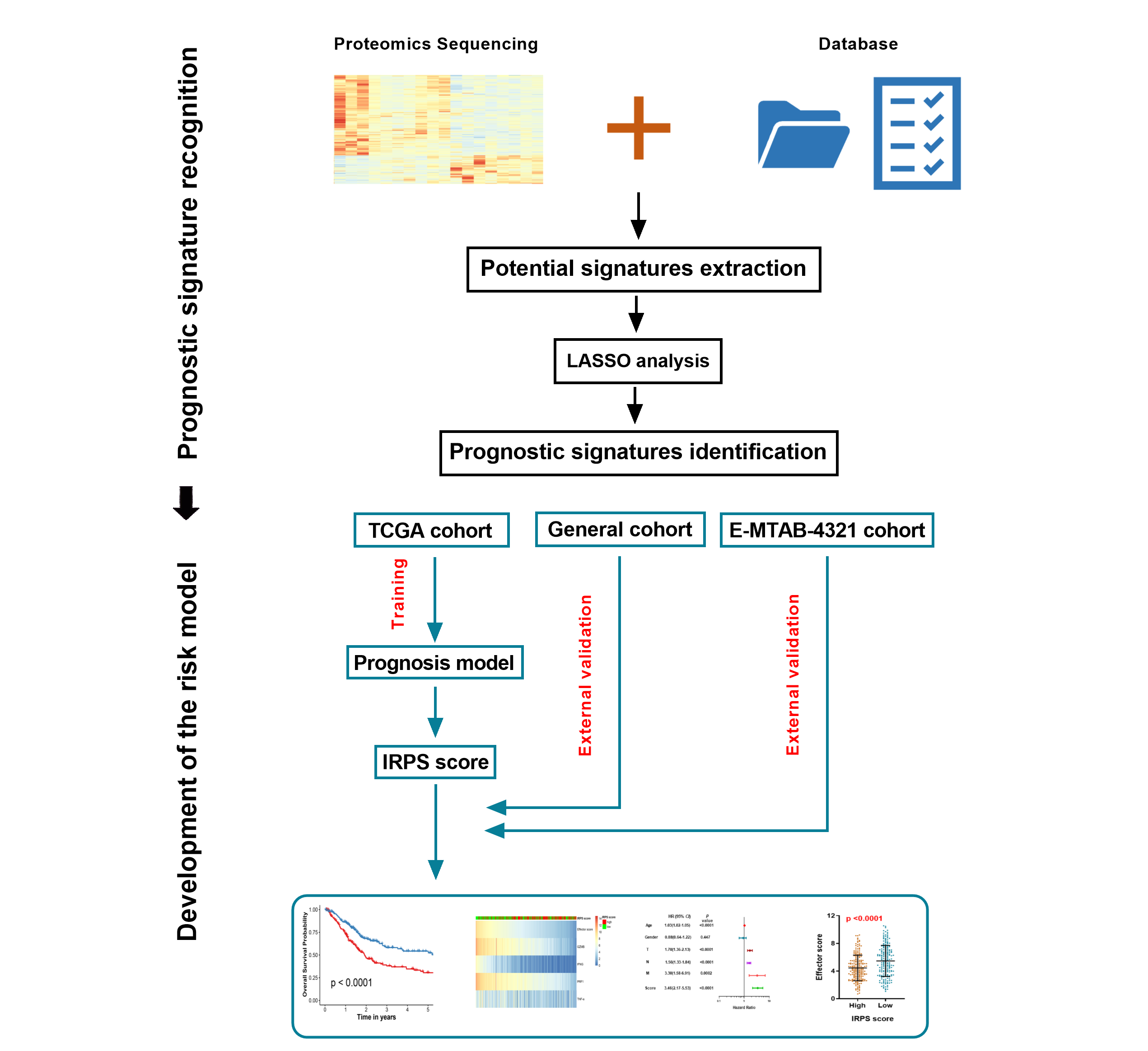

Supplement: Supplementary file 1 — Additional file 1. The workflow of the study. [file 12885_2022_9783_MOESM1_ESM.tif]

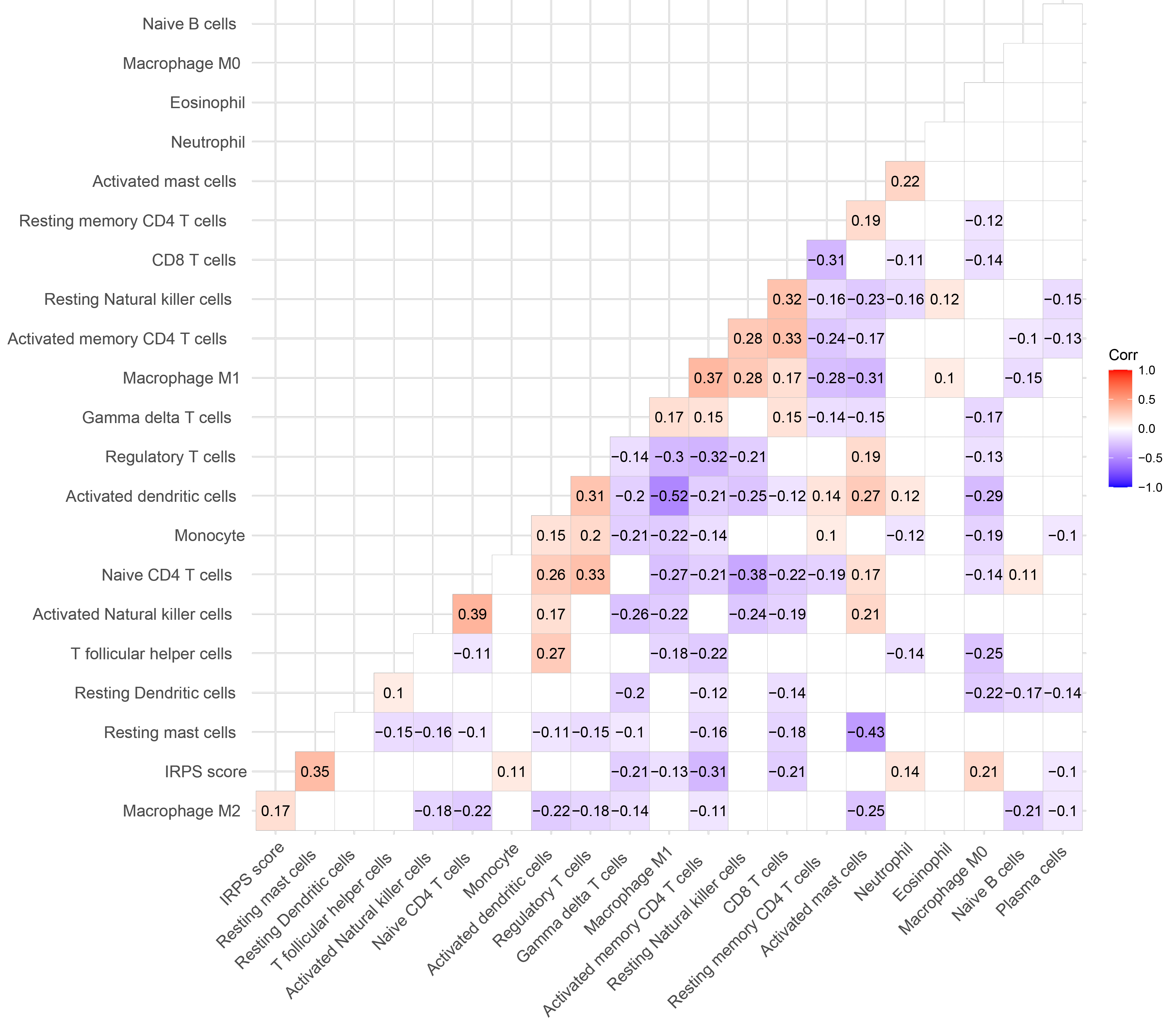

Supplement: Supplementary file 2 — Additional file 2. Correlation analysis of IRPS score and immune infiltration cells. [file 12885_2022_9783_MOESM2_ESM.tif]
